# Measles Virus Neutralizing Antibody Response and Durability Two Years after One or Two Doses of Measles–Mumps–Rubella Vaccine among Young Seronegative Healthcare Workers

**DOI:** 10.3390/vaccines10111812

**Published:** 2022-10-28

**Authors:** Byungki Jang, Han Wool Kim, Han-Sung Kim, Ji Young Park, Hyeonji Seo, Yong Kyun Kim

**Affiliations:** 1 Ilsong Institute of Life Science, Hallym University, Seoul 01000, Korea; 2 Department of Pediatrics, Hallym University Sacred Heart Hospital, Hallym University College of Medicine, Anyang 14100, Korea; 3 Department of Laboratory Medicine, Hallym University Sacred Heart Hospital, Hallym University College of Medicine, Anyang 14100, Korea; 4 Department of Internal Medicine, Division of Pulmonary, Allergy and Critical Care Medicine, Hallym University Sacred Heart Hospital, Hallym University College of Medicine, Anyang 14100, Korea; 5 Department of Internal Medicine, Division of Infectious Diseases, Hallym University Sacred Heart Hospital, Hallym University College of Medicine, Anyang 14100, Korea

**Keywords:** measles, measles–mumps–rubella vaccine, healthcare workers, immune durability, plaque reduction neutralization test, neutralizing antibody

## Abstract

Although there have been several studies regarding the immunogenicity of one or two booster doses of the measles–mumps–rubella (MMR) vaccine in measles-seronegative young adults, limited data are available about how long the immune response is sustained compared with natural infection. This study included seronegative healthcare workers (HCWs) (aged 21–38 years) who received one or two doses of the measles–mumps–rubella (MMR) vaccine and HCWs with laboratory-confirmed measles infection during an outbreak in 2019. We compared neutralizing antibody titers measured using the plaque reduction neutralization (PRN) test and measles-specific immunoglobulin G (IgG) using chemiluminescent immunoassays 2 years after vaccination or infection. Among 107 HCWs with seronegative measles IgGs, the overall seroconversion rate of measles IgGs remained 82.2% (88/107), and 45.8% (49/107) of the participants had a medium (121–900) or high (>900) PRN titer after 2 years from one or two booster doses. The measles-neutralizing antibody titers of both PRN titer (ND50) and geometric mean concentration 2 years after natural infection were significantly higher than those of one or two booster doses of the MMR vaccine (*p* < 0.001 and *p* < 0.001, respectively). Our results suggest that serologic screening followed by appropriate postexposure prophylaxis can be beneficial for young HCWs without a history of natural infection especially in a measles outbreak setting, because of possible susceptibility to measles despite booster MMR vaccination 2 years ago. Long-term data about sustainable humoral immunity after one or two booster vaccination are needed based on the exact vaccination history.

## 1. Introduction

The extremely high transmissibility of the measles virus caused its worldwide resurgence between 2017 and 2019 [1]. In recent measles outbreaks, many adolescents and young adults were found to be incompletely vaccinated [2]; however, an increase in the measles-susceptible adult population has also been identified in countries with high vaccination rates and good measles control [3,4]. Although the receipt of two doses of the measles–mumps–rubella (MMR) vaccine has been accepted as proof of immunity, secondary vaccine failure with waning neutralizing antibody levels and a limited extent of natural boosting may account for this phenomenon [5].

Pockets of lower immunity against measles in young healthcare workers (HCWs) aged 20–30 years have been observed [6,7,8], causing particular concern due to the possibility of a nosocomial measles outbreak [9,10]. The vaccination policy against measles for HCWs has been increasingly strengthened as a pre-requisite for new employment [11], and the administration of a third dose of the MMR vaccine (MMR3) to young HCWs is being considered in Korea [12,13].

The current Advisory Committee on Immunization Practices guideline does not recommend that individuals receive an additional dose of MMR vaccine if they have prior 2 documented doses of MMR vaccine [14]. However, documented receipt of two doses of MMR does not guarantee immunity to HCWs who are vulnerable to both infection and vehicles of onward transmission [9,10] because measles antibody seronegativity, regardless of vaccination history, may have a significant correlation with measles infection [4]. Recently, there have been several studies regarding the immunogenicity of one or two booster doses of MMR vaccine in measles-seronegative young adults who were vaccinated in their childhood [15,16,17,18]. However, those studies only showed the short-term development of the antibody response [15,16,17,18]; limited objective data are available about how long the immune response is sustained after one or two booster doses of the MMR vaccine in young, measles-seronegative HCWs.

Therefore, in this study, we aimed to evaluate the immune response and durability 2 years after one or two booster MMR vaccine doses in young, seronegative HCWs. In addition, we also aimed to compare the durability of neutralizing antibody levels 2 years after one or two booster doses of MMR vaccine in young, seronegative HCWs versus that 2 years after natural infection of the measles virus.

## 2. Materials and Methods

### 2.1. Study Population and Clinical Data Collection

This study was conducted at Hallym University Sacred Heart Hospital, an 829-bed, a university-affiliated hospital in Anyang, Korea. At this hospital, a nosocomial measles outbreak occurred in 2019, and emergent serological testing was required for measles immunity verification for all HCWs, followed by one or two doses of the MMR vaccine for seronegative HCWs as infection control measures [19].

The cohorts included measles-specific IgG-negative HCWs (aged 21–38 years) who received one or two doses of the MMR vaccine during the outbreak in 2019. Cohort 1 (*n* = 84) patients received one dose of the MMR vaccine (PRIORIX; GlaxoSmithKline, Belgium) and were found to have seroconverted 4 weeks later in the second serological testing in 2019. Cohort 2 (*n* = 23) received the second dose of the MMR vaccine (PRIORIX; GlaxoSmithKline) 4 weeks after the first dose because they remained measles-seronegative at the second serological testing in 2019. Serum samples were collected from both cohorts to assess neutralizing antibody and measles-specific IgG levels 2 years after vaccination (June 2021). Additionally, 22 HCWs exhibited laboratory-confirmed measles infection during the outbreak in 2019. We obtained the serum samples of 12 such HCWs (Cohort 3) to determine their neutralizing antibody and measles-specific IgG levels 2 years after infection (June 2021).

The subjects that were not seroconverted in June 2021 despite the administration of two doses of the MMR vaccine in 2019 were considered non-responders. The documented vaccination records of the HCWs enrolled in this study were obtained from the computerized and integrated vaccination registration data of the Korea Disease Control and Prevention Agency.

### 2.2. Plaque Reduction Neutralization Test

Vero cells (Korean Cell Line Bank, Korean Cell Line Research Foundation, Seoul, Korea) were seeded at 1 × 10^5^ cells/500 μL media/well in 24-well culture plates in Eagle’s minimum essential medium (EMEM; ATCC, Manassas, VA, USA) containing 10% fetal bovine serum (FBS; Biowest, Riverside, MO, USA) and antibiotics (Thermo Fisher Scientific, Waltham, MA, USA) and cultured for one day at 37 °C in a 5% CO_2_ incubator. Then, 50 μL of test serum and 20 μL of 3rd international standard (3rd IS; NIBSC, code: 97/648) for anti-measles were incubated for 30 min at 55 °C. The test serum and 3rd IS were then diluted to 1/4 and 1/16 of the original concentrations, respectively, with four-fold serial dilutions in 96-well plates. All chemicals and reagents were purchased from Sigma-Aldrich (St. Louis, MO, USA), and measles virus (GenBank Sequence Accession: K01711) was obtained from the American Type Culture Collection (Edmonston strain, VR-24, ATCC, Manassas, VA, USA). An equal volume of diluted Edmonston measles virus was added to each well. The final virus dilution was 1:1400, and the test serum and 3rd IS dilutions began at 1/8 and 1/32, respectively. After the virus and virus-serum mixtures were incubated for 2 h in a CO_2_ incubator, 50 μL of the virus or virus-serum mixture was added to the Vero cells (culture medium-aspirated), along with 450 μL of the culture medium (2% FBS/antibiotics/EMEM). After the cells were incubated for 90 min, 400 μL of culture medium was added, followed by 300 μL of 3.2% carboxymethylcellulose sodium salt (CMC) overlay medium (dissolved in EMEM). After 6 days of incubation, the medium containing CMC (Merck, Darmstadt, Germany) overlay was removed, and the cell monolayers were fixed with 10% formaldehyde (Junsei Chemical, Tokyo, Japan) for 30 min and stained with 1% crystal violet (Sigma, Yongin, Korea) for 30 min. Finally, the plates were rinsed with 1 mL of distilled water and allowed to air dry. The 50% end-point titers (neutralizing dose, ND50) were calculated using the Kärber formula [20], and ND50 results were transformed into geometric mean titers (GMTs, mIU/mL) as previously described [20,21,22]. According to previous studies, the plaque reduction neutralization test (PRNT) results were categorized as negative for measles neutralizing antibody (<8), low (8–120), medium (121–900), or high (>900) [15,23,24]. Negative or low PRNT results indicated potential susceptibility, and medium or high PRNT results indicated non-susceptibility to measles infection [15,23,24].

### 2.3. Chemiluminescent Immunoassay for Measles-Specific IgGs Assay

To determine the measles-specific IgG levels, all test sera were assessed using LIAISON Measles IgG chemiluminescent immunoassay (CLIA) as described in the manufacturer’s instructions (DiaSorin, Saluggia, Italy). The positive cut-off value was ≥16.5 arbitrary units (AU)/mL, according to the manufacturer’s protocol.

### 2.4. Statistical Analysis

Categorical variables were expressed as frequencies and proportions and compared using the χ^2^ or Fisher’s exact test. Continuous variables were expressed as median values with interquartile ranges and compared using the Mann–Whitney U test, as appropriate. Geometric mean antibody titers with 95% confidence intervals (CIs) were calculated from the PRN titers (ND50) using log-transformed values as the primary analysis measures for each group. All tests of significance were two-tailed, and *p*-values < 0.05 were considered to be statistically significant. All data were analyzed using IBM SPSS Statistics for Windows, version 21 (IBM Corp., Armonk, NY, USA).

## 3. Results

### 3.1. Enrollment and Baseline Characteristics

**Figure 1:**
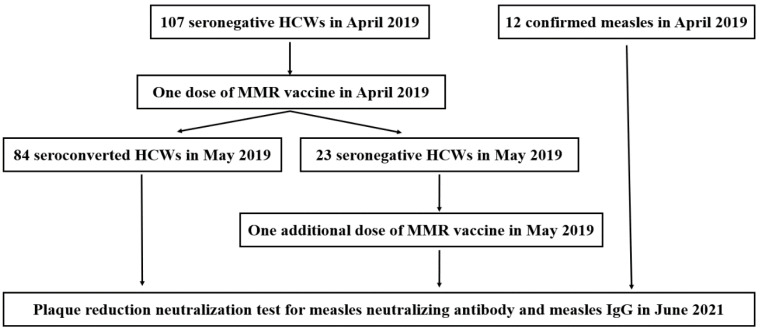
Study flow diagram.

A total of 107 seronegative HCWs who received one (Cohort 1, *n* = 84) or two (Cohort 2, *n* = 23) doses of the MMR vaccine and 12 HCWs (Cohort 3) with laboratory-confirmed measles infection were enrolled in the present study (Figure 1). The differences in age and documented vaccination history are presented in Table 1. The median (interquartile range [IQR]) ages were 27 (24–31) and 24 (23–26) in Cohorts 1 and 2, respectively, which showed no significant difference (*p* = 0.12). In Cohort 1, 55 HCWs (65%) had documentation of MMR vaccination prior to the outbreak in 2019 (47/84, 56% with prior receipt of one dose; 8/84, 10% with prior receipt of two doses). In Cohort 2, 14 HCWs (61%) had documentation of one prior dose of MMR vaccination, and no HCWs had documentation of two doses. In Cohort 3, the median (IQR) age was 25 (24–27) years, and 11 HCWs (92%) had documentation of prior MMR vaccination (25% [3/12] received one dose and 67% [8/12] received two doses). Of the 119 HCWs enrolled in the study, 64 had documented receipt of one dose of MMR vaccine prior to antibody sampling (47 in Cohort 1, 14 in Cohort 2, and 3 in Cohort 3). The median time from the last MMR vaccine to antibody sampling was 18 years (IQR: 18–19 years), 18 years (IQR: 17–19 years), and 18 years (IQR: 18–19 years) in Cohorts 1, 2, and 3, respectively, did not differ significantly between the cohorts (*p* = 0.41 in Cohort 1 vs. Cohort 2; *p* = 0.39 in Cohort 1 vs. Cohort 3). In addition, 16 HCWs had documented receipt of two MMR doses prior to antibody sampling (8 in Cohort 1 and 8 in Cohort 3). The median age at the time of the first dose of the MMR vaccine (60 months; IQR: 32–78 months vs. 32 months; IQR: 12–73 months, *p* = 0.27), and of the second dose of the MMR vaccine (7 years; IQR: 6–9 years vs. 7 years; IQR 5–9 years) did not differ significantly (*p* = 0.66). The median time from the first to the second MMR vaccine (15 months; IQR 4–39 months vs. 30 months; IQR 23–61 months, *p* = 0.18), from the first MMR vaccine to antibody sampling (19 years; IQR: 18–22 years vs. 21 years; IQR: 19–23 years, *p* = 0.33), and from the second MMR vaccine to antibody sampling (18 years; IQR: 18–18 years vs. 21 years; IQR: 17–18 years) did not differ between Cohort 1 and Cohort 3 (*p* = 0.67).

### 3.2. Measles Neutralizing Antibody Response 2 Years after One or Two Doses of MMR Booster

**Figure 2:**
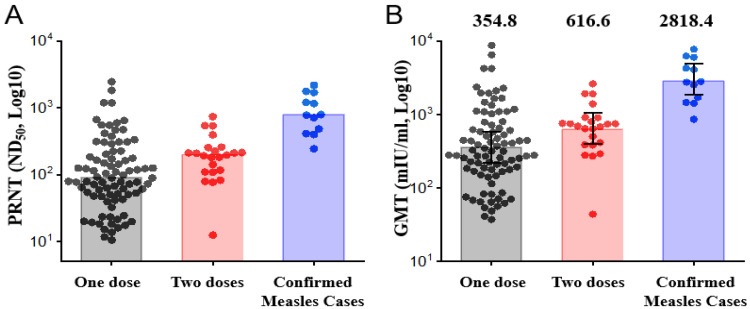
Measles virus neutralizing antibody titers in vaccinated and infected HCWs: (**A**) PRNT ND50 values of one or two MMR doses and confirmed measles cases are represented by a dot plot and bars. The heights of the bars indicate the median value. (**B**) Individual neutralizing antibody titers are shown as a dot plot, and the bars represent the geometric mean titers, with error bars that show 95% confidence intervals. The values above the bars are the geometric mean titers.

The results of PRNT with Vero cells to assess the measles virus-neutralizing antibody titers of the 107 enrolled HCWs are presented in Table 2 and Figure 2. The ND50 after 2 years in Cohort 1 (median: 88.6; IQR: 47.0–196.8) was significantly lower than that in Cohort 2 (median: 195.2; IQR: 108.8–253.3; *p* = 0.007). The cut-off value of above 120, indicating non-susceptibility to measles infection, was observed in 33 (33/84, 39%), 16 (16/23, 70%), and 12 (12/12, 100%) HCWs in Cohorts 1, 2, and 3, respectively. A low PRN titer of measles-neutralizing antibodies (8–120) was significantly more common in Cohort 1 and Cohort 2 than in Cohort 3 (54%, 58/107 vs. 0%, 0/12, *p* < 0.001). The ND50 of Cohort 3 was above the threshold of non-susceptibility to measles infection (>120), indicating that natural infection was effective in preventing measles infection. Analysis of the GMTs derived from PRN titers, expressed in mIU/mL (mean conversion factor 4.02, ranging from 1.91 to 7.39), after 2 years the GMT in Cohort 1 (mean: 354.8 mIU/mL; 95% CI: 273.8–459.8 mIU/mL) was significantly lower than that in Cohort 2 (mean: 616.6 mIU/mL; 95% CI: 438.6–866.9 mIU/mL; *p* = 0.009). The ND50 (median: 113.6; IQR: 54.3–223.5) and the GMTs derived from PRN titers (mean: 398.1 mIU/mL; 95% CI: 319.5–496.0 mIU/mL) after 2 years in Cohort 1 and Cohort 2 were significantly lower than those in Cohort 3 (median: 779.8; IQR: 426.3–1558.9; *p* < 0.001 and mean: 2818.4 mIU/mL; 95% CI: 1917.3–4143.1 mIU/mL; *p* < 0.001, respectively).

### 3.3. Measles-Specific IgGs Response 2 Years after One or Two Doses of the MMR Vaccine

The measles-specific IgG levels of the 107 enrolled HCWs are presented in Table 3. The overall seroconversion rate 2 years after one or two booster doses of the MMR vaccine was 82.2% (88/107), not significantly different from that of natural infection (82.2% vs. 100%, *p* = 0.21). In Cohort 1, 12 HCWs (12/84, 14%) had seroconverted from positive to negative 2 years after one MMR vaccine dose. However, measles IgGs seropositivity was significantly lower in Cohort 2 than in Cohort 1 (14/23, 61% vs. 72/84, 86%, *p* = 0.008), whereas Cohort 3 exhibited 100% positivity of measles-specific IgGs.

## 4. Discussion

This study presents the immune response and durability 2 years after one or two doses of booster MMR vaccination or natural infection in young, seronegative HCWs. The results showed that 54.2% (58/107) of HCWs who received one or two booster doses of MMR vaccine had a low PRN titer of measles neutralizing antibodies (8–120) after 2 years, while the overall seroconversion rate of measles IgGs was 82% (88/107). The measles-neutralizing antibody titers of both PRN titer (ND50) and geometric mean concentration after 2 years of natural infection were significantly higher than those of one or two booster doses of the MMR vaccine.

In the post-elimination era, vaccine-induced measles immunity in the absence of natural boosting may cause waning immunity, which results in protective antibody persistence after the second dose of only 10–15 years, rather than a lifetime [25,26,27,28]. This finding highlights the need for an additional dose of the MMR vaccine in young adults, especially in high-risk populations, such as HCWs. Several recent studies on the measles virus antibody response of the booster MMR vaccine support its administration to young, measles-seronegative adults [15,16,17,18]; in these studies, medium (121–900) or high (>900) PRN titers were observed in 75% (18/24) of the participants one month after a third dose of the MMR vaccine in the low or negative baseline PRN titer groups [15], while the overall seroconversion rates of measles IgGs were 89.7 to 98.9% 4 weeks after two booster doses [16,17,18]. In addition, several studies evaluated the short-term persistence of measles antibodies, finding a range of 6 months to 1 year. Fiebelkorn et al. [15] reported the one-year immunogenicity of a third MMR vaccine dose among 20 young adults with low or negative measles-neutralizing antibody levels at baseline, which observed that the measles-neutralizing antibody levels of 15 subjects (75%) remained medium or high after one year. Seok et al. [29] reported measles virus neutralizing antibodies 6 months after two doses of the MMR vaccination in 55 seronegative HCWs, which revealed 100% of medium (121–900) or high (>900) PRN titers. Kaaijk et al. [30] compared the antibody response of two MMR vaccine doses with that of a third dose of the MMR vaccine. In their study, 3% of 147 young adults (18–25 years of age) had no seroprotection at baseline, and the seroprotection rate remained 100% above the threshold up to one year after a third dose of the MMR vaccine.

Compared with previous studies, we observed the immune response to one or two additional MMR vaccine doses persists over a relatively long period of 2 years. Our study is strengthened by the relevant number of seronegative young adults (*n* = 107) at baseline, and we performed PRNT, a gold standard method to detect measles-neutralizing antibodies for all of the study subjects. Furthermore, we compared the durability of measles-neutralizing antibodies in young, seronegative HCWs who received one or two booster MMR vaccines with that of HCWs with natural infections.

Our study suggests that only 45.8% (49/107) of the HCWs who received one or two booster doses of the MMR vaccine had a medium (121–900) or high (>900) PRN titer of measles neutralizing antibodies after 2 years, suggesting that booster doses in young, seronegative adults may not confer sustainable humoral immunity. It has been poorly studied whether the dynamics of the antibody response after MMR vaccination in young adults differ from those in children and whether neutralizing antibody titers after MMR vaccination in young, seronegative adults can be sustained through adulthood. Kaaijk et al. [31] reported that all study subjects (aged 18–25) had clinical protective levels of measles antibodies after 3 years of a third dose of the MMR vaccine. However, 97% of the study subjects were already seropositive prior to the booster MMR dose in that study, which made it difficult to evaluate the 3-year immune durability of booster doses in young, seronegative adults. Kennedy et al. [32] suggested that measles vaccine-induced immunity might wane or drop relatively early, within 7 years post-vaccination, in school-aged children. Taken together, our results support the hypothesis that the immunogenicity of the MMR vaccine in young, seronegative adults may be less durable and characterized by more rapid waning. However, we believe that our results do not imply that booster vaccination in young adults is unnecessary, as T-cell immunity has an important role in the development of immunity against measles [33,34]. In addition, we posit that our data are insufficient to recommend two routine booster doses rather than one dose because the baseline vaccination history of our study cohorts varied considerably. Although our results showed that one booster of the MMR vaccine in seronegative young adults might be associated with a more rapid decline of measles neutralizing antibody levels compared with two booster doses, a cautious interpretation of our results is needed until more well-designed studies are performed. Future studies should include a large number of young, seronegative adults who received the MMR vaccination twice and should evaluate both neutralizing antibodies and cellular immunity after booster doses. We believe that our study has important clinical implications for further well-designed research for a better understanding of the long-term persistence of protective immune response to measles.

Our study suggests that the 2-year immune response and durability of one or two booster doses of MMR vaccine is reliable, given the overall seroconversion rate of 82.2%. However, the antibody response after 2 years of one or two booster doses of the MMR vaccine was significantly lower than that of natural infection, and some HCWs without a history of natural infection may be susceptible to measles virus infection despite one or two booster MMR vaccination 2 years ago. Therefore, we advocate measles serology test with appropriate postexposure prophylaxis for young HCWs without a history of natural infection, especially in measles outbreak setting. We believe that our study has important implications for future study to establish an optimal strategy for control of nosocomial measles outbreak. Basically, we support the routine serologic screening test of measles IgGs for young HCWs at new employment when their last MMR vaccination occurred 10–15 years ago, which can be reinforced by previous studies regarding the immunogenicity of a booster dose in young, seronegative adults [15,16,17,18,29,30]. The current guideline does not recommend an additional dose of MMR vaccine if there is documented receipt of two doses of MMR vaccine [14]. However, waning vaccine-induced immunity over time is associated with increased measles seronegativity and susceptibility [4], and there have been reports of measles cases who received 2 doses of MMR but had negative measles IgGs [35,36] or measles outbreaks among individuals with documented receipt of prior two doses of MMR vaccine [37,38]. In addition, the rates of seronegative HCWs despite the documented receipt of prior 2-dose vaccination was up to 15% in a recent study with a relevant sample size [17,39], and the rate of measles-susceptibility was substantially high in young HCWs in the post-elimination setting [8,18,19,40,41,42]. Therefore, we advocate for a policy of routine serologic screening, followed by one or two booster doses of the MMR vaccine for young, seronegative HCWs, especially for those that work in departments with a high risk of exposure or immunocompromised patients. We assume that this strategy may be cost-effective, as previous studies demonstrated that the pre-vaccination assessment of highly contagious diseases for HCWs was cost-effective [43,44]. The HCWs with a proven history of natural infection could be an exception to routine screening because the scientific evidence to date, including this present study, showed a better immune durability of natural infection compared with vaccine-induced immunity [29,45,46]. Further studies to evaluate the cost-effectiveness of this strategy may be warranted, as financial support is the key element for the implementation of the appropriate hospital vaccination policy [47]. The incidence and severity of adverse reactions after MMR vaccine in young HCWs are also important issues [48,49], which should be evaluated in future studies.

Our study has several limitations. First, our study was not prospectively designed and we did not estimate sample size calculation for optimal statistical power. Second, not all of the unrecorded data of MMR vaccination history could be obtained, as records in the national computerized and integrated database on vaccine registration of the Korea Disease Control and Prevention Agency could be missing. In addition, we did not include memory-dependent methods to improve the objectivity of MMR vaccination history. In South Korea, after a large outbreak during 2000–2001 affecting 55,696 individuals, a measles-rubella catch-up vaccination was performed in 2001 on those born between 1985 and 1994. In addition, a law required those born after 1994 to receive a routine second dose of the MMR vaccine and submit the certificate of vaccination before entering elementary school in 2005 [50]. Future studies including a cohort of fully vaccinated, measles-seronegative young adults are necessary to evaluate the immunogenicity or immune durability of MMR3 or MMR4 more accurately and can be more informative if previous vaccination history is considered in the analysis of determinants of seroconversion after one or two booster MMR doses. Third, we did not collect samples serially between one or two booster vaccinations and 2 years after the last booster vaccination, hindering the evaluation of the dynamics of the antibody response. Fourth, we could not investigate the risk factors of non-responders and cellular immune response to the MMR booster vaccine. The non-responsiveness can affect about 2–10% of vaccinated healthy HCWs [51], and we observed nine non-responders (seronegative individuals in cohort 2 even after the second vaccine dose) in our study. Although the underlying mechanism of non-responsiveness of MMR vaccination is not certain, genetic predisposition may affect vaccine failure [52]. However, cellular immunity induced by the measles vaccine may protect vaccinated subjects, despite the hypo-responsiveness at the humoral level [51,53]. Future studies to evaluate the immunological characteristics responsible for non-responsiveness in risk populations such as HCWs and develop specific recommendations in case of exposure are necessary to help in the decision-making process in hospital infection control. Finally, we used a CLIA to measure seroprevalence in this study. Previous studies showed that the CLIA used in this study showed an acceptable performance in detecting measles IgGs [54,55], and had a sensitivity of 97.2% and a specificity of 92.9% compared with the Enzygnost anti-measles IgG test (Siemens Healthcare Diagnostics Products GmbH, Marburg, Germany) [54]. However, our seroprevalence results should be interpreted with caution, and we posit that further validation with well-designed studies to determine its ability to detect measles immunity compared with PRNT should be conducted. Despite these limitations, we believe that our study is valuable as it evaluated the 2-year persistence of measles-neutralizing antibodies in HCWs who received one or two booster doses of MMR vaccine and with natural infection, using the gold standard assay in a significant number of seronegative young adults at baseline. This study has important clinical implications for further well-designed research to create an optimal vaccination policy for measles-seronegative HCWs.

## 5. Conclusions

Our findings that the overall 2-year seroconversion rate of measles IgG after one or two booster doses of MMR vaccine (82%, 88/107) and decreased PRN titers in young seronegative HCWs suggest that measles serology test with appropriate postexposure prophylaxis for HCWs without a history of natural infection can be helpful as a response to a measles outbreak. Long-term data about sustainable humoral immunity are needed because only half of young, seronegative HCWs with one or two booster doses of MMR vaccine had a medium or high PRN titer of measles-neutralizing antibodies after 2 years.

## Figures and Tables

**Table 1:** Demographic of each cohort of one or two booster doses of MMR vaccine or laboratory-confirmed measles infection among young, seronegative healthcare workers.

Variables	One Dose (*n* = 84)	Two Doses (*n* = 23)	Confirmed Case (*n* = 12)
Age, median years	27 (24–31)	24 (23–26)	25 (24–27)
Male	22 (26.2)	19 (82.6)	3 (25.0)
Documented measles vaccination history before study enrollment			
Unknown	29 (34.5)	9 (39.1)	1 (8.3)
1 dose received	47 (56.0)	14 (60.9)	3 (25.0)
2 doses received	8 (9.5)	0 (0)	8 (66.7)
Time from the last MMR vaccine to antibody sampling, years	18 (18–19)	18 (17–19)	18 (18–19)
Age at the time of first MMR vaccine, months	60 (32–78)	NA	32 (12–73)
Age at the time of second MMR vaccine, years	7 (6–9)	NA	7 (5–9)
Time from the first to the second MMR vaccine, months	15 (4–39)	NA	30 (23–61)
Time from the first MMR vaccine to antibody sampling, years	19 (18–22)	NA	21 (19–23)
Time from the second MMR vaccine to antibody sampling, years	18 (18–18)	NA	18 (17–18)

Data are presented as numbers (%) or median (interquartile range) unless indicated otherwise.

**Table 2:** Comparison of two-year immune response and immune durability of each group after one or two booster doses of MMR vaccine or laboratory-confirmed measles infection among young, seronegative healthcare workers.

Variables	Booster Dose			Confirmed Case (*n* = 12)	*p* Value ^a^	*p* Value ^b^
	One Dose (*n* = 84)	Two Doses (*n* = 23)	Total (*n* = 107)			
PRNT, ND50	88.6 (47.0–196.8)	195.2 (108.8–253.3)	113.6 (54.3–223.5)	779.8 (426.3–1558.9)	0.007	<0.001
Low (8–120)	51 (60.7)	7 (30.4)	58 (54.2)	0 (0)	0.010	<0.001
Medium (121–900)	29 (34.5)	16 (69.6)	45 (42.1)	7 (58.3)	0.003	0.31
High (>900)	4 (4.8)	0 (0)	4 (3.7)	5 (41.7)	0.58	<0.001
GMT (mIU/mL), mean (95% CI)	354.8 (273.8–459.8)	616.6 (438.6–866.9)	398.1 (319.5–496.0)	2818.4 (1917.3–4143.1)	0.009	<0.001

PRNT, plaque reduction neutralization test; ND50, neutralizing dose 50; CI, confidence interval; GMT, geometric mean titer. Data are presented as numbers (%) or median (interquartile range) unless indicated otherwise. ^a^ One booster dose vs. two booster doses. ^b^ One or two booster doses vs. confirmed case.

**Table 3:** Comparison of measles-specific IgG response of each group after one or two booster doses of MMR vaccine or laboratory-confirmed measles infection among young, seronegative healthcare workers.

Variables	Booster Dose			Confirmed Case (*n* = 12)	*p* Value ^a^	*p* Value ^b^
	One Dose (*n* = 84)	Two Doses (*n* = 23)	Total (*n* = 107)			
Measles IgG positive (CLIA)	72 (85.7)	14 (60.9)	88 (82.2)	12 (100)	0.008	0.21

CLIA, chemiluminescent immunoassay. Data are presented as numbers (%) unless indicated otherwise. ^a^ One booster dose vs. two booster doses. ^b^ One or two booster doses vs. confirmed case.

## Data Availability

The data are available upon reasonable request by contacting the corresponding author.
